# Association of sex differences in dementia risk factors with sex differences in memory decline in a population-based cohort spanning 20–76 years

**DOI:** 10.1038/s41598-021-86397-7

**Published:** 2021-04-08

**Authors:** Kaarin J. Anstey, Ruth Peters, Moyra E. Mortby, Kim M. Kiely, Ranmalee Eramudugolla, Nicolas Cherbuin, Md Hamidul Huque, Roger A. Dixon

**Affiliations:** 1 School of Psychology, University of New South Wales, Sydney, Australia; 2 Neuroscience Research Australia, 139 Barker Street, Randwick, NSW 2031 Australia; 3 Centre for Research on Ageing Health and Wellbeing, School of Population Health, The Australian National University, Canberra, Australia; 4 Department of Psychology, University of Alberta, Edmonton, Canada; 5 Neuroscience and Mental Health Institute, University of Alberta, Edmonton, Canada

**Keywords:** Neurological disorders, Psychiatric disorders, Psychology, Risk factors

## Abstract

Sex differences in late-life memory decline may be explained by sex differences in dementia risk factors. Episodic memory and dementia risk factors were assessed in young, middle-aged and older adults over 12 years in a population-based sample (N = 7485). For men in midlife and old age, physical, cognitive and social activities were associated with less memory decline, and financial hardship was associated with more. *APOE e4* and vascular risk factors were associated with memory decline for women in midlife. Depression, cognitive and physical activity were associated with memory change in older women. Incident midlife hypertension (β = − 0.48, 95% CI − 0.87, − 0.09, p = 0.02) was associated with greater memory decline in women and incident late-life stroke accounted for greater memory decline in men (β = − 0.56, 95% CI − 1.12, − 0.01), p = 0.05). Women have fewer modifiable risk factors than men. Stroke and hypertension explained sex differences in memory decline for men and women respectively.

## Introduction

Recent discussion has called for sex-based investigation of the aetiology of dementia^1^, given meta-analyses have shown that Alzheimer’s disease (AD) is more prevalent in women^2^. A review of data from Europe found that the prevalence of AD was 7.02 per 1000 person-years in men and 13.25 per 1000 person-years in women^2^. In comparison, Vascular dementia is more prevalent in men (5.6 cases versus 3.2 cases per 1000)^3^. However, these differences in dementia prevalence are contradicted by other research in the field. For example, studies have found that in cognitively normal samples, women have better performance on measures of episodic memory in late life^4^, even though women are more likely to develop AD which is characterised typically by memory loss. A critical question for understanding sex differences in the prevalence of AD and dementia more broadly, is the extent to which sex differences in the prevalence and incidence of AD and dementia can be attributed to sex differences in dementia risk factors, and to sex differences in the strength of the association between risk-factors and dementia^5^. Preceding dementia, the same question can be asked of the relationship between dementia risk factors and cognitive decline more broadly.

Recent reviews have identified a wide range of risk factors for dementia^6^ and studies have shown that there is a cumulative effect of increasing numbers of risk factors^7^. It is possible that combinations or patterns of risk factors explain sex differences in cognitive decline and subsequent risk of dementia. Therefore, a multi-domain approach to understanding and analysing risk factors is arguably the best approach for investigating sex differences in dementia risk and also memory decline. Broad domains of risk factors have been previously classified as biomarkers, demographic variables, lifestyle factors, medical conditions and medications, and environmental factors^8^. In addition, there is broad recognition that a life-course approach is needed to fully understand the role of risk factors for cognitive decline and dementia^9^.

Life-course studies including the same memory measures repeated over time in different age cohorts provide information on when sex differences in memory begin to emerge, or to detect the factors that are associated with this emergence. Understanding sex differences in specific risk factors and their association with memory decline may help with earlier detection and intervention, and possibly developing tailored risk reduction approaches. Without longitudinal data it is not possible to evaluate whether the sex differences in dementia are associated with earlier sex differences in memory decline and the factors that sustain them.

The motivation for this study is to evaluate sex differences in dementia risk factors and identify whether they explain sex differences in memory performance and memory decline. Risk factors were identified from previous literature reviews^6,10–12^. Our specific aims are to (a) characterise how men and women differ in memory performance and decline from early to late adulthood, and (b) determine whether sex differences in change in risk factors over time explain the sex differences in memory change.

## Methods

### Participants and sample information

The data were drawn from the Personality and Total Health Through Life (PATH) Project^13^. At Wave 1 (baseline) participants were aged 20–24 (1162 men and 1242 women; 91% Caucasian), 40–46 (1337 women and 1193 men; 94% Caucasian) and 60–64 (1289 men and 1220 women 96% Caucasian)^14,15^. Participants were randomly sampled from the electoral roll of the Australian Capital Territory and Queanbeyan in Australia. Voting is compulsory in Australia. Participants were followed up three times at intervals of approximately four years. The age-range was 20–76. The dataset includes information on health and lifestyle, social relationships, medical conditions, and genotyping. Written informed consent was obtained and approval from the Human Ethics Committee of The Australian National University.

After 12 years, 61.8% (*n* = 4597) of the original sample was retained [excluding those who had died (5%), moved or were lost to follow-up (25.7%)]. Details are provided in Supplementary Material Table A1. At each wave, demographic, lifestyle, mental health, medication, and health measures were obtained through a self-completed survey. Self-report data was obtained through validated scales where possible and the wording of questions kept consistent across the waves and cohorts. Physical function and cognitive assessment were obtained during an in-person interview. For this study 42 participants from the oldest cohort who scored < 24 on the Mini-Mental State Examination at baseline were excluded due to likely dementia. Due to funding limitations a random subsample of the 20 s cohort had cognitive assessments at Wave 4 (n = 546). Measures included in this study are described according to five domains including biomarkers, socio-demographic, lifestyle, medical, environmental^12^.

### APOE status

The biomarker included in this study was *APOE* genotype. Genotyping of the PATH sample has been previously described^16^. Briefly, genomic DNA was extracted from buccal swabs using Qiagen Blood kits. Two TaqMan assays were performed to ascertain the genotypes of the two SNPs defining the *APOE* alleles, rs429358 and rs7412. Overall, 95.3% of the 20 s cohort, 90.6% of the 40 s cohort, and 90.1% of the 60 s cohort provided buccal swabs. Genotype frequencies did not deviate from the Hardy–Weinberg equilibrium. After removing participants carrying the *APOE e2/e4* genotype, *APOE* genotype status was coded into three categories (*E4*+*/E4−*, *E4*+*/E4*+, or *E4−/E4−*).

### Socio-demographic domain

Level of education was the total years of self-reported primary, secondary and post-secondary, or vocational education. English as a second language (yes/no) was obtained at baseline, as was ethnicity (Caucasian/white, Asian, other). Self-reported marital or de facto relationship status and whether participants had to go without basic needs being met in the past year due to financial hardship (Financial hardship: yes/no) were obtained at each wave. Self-reported childhood financial hardship was reported as part of a childhood adversity scale^17^.

### Lifestyle domain

Lifestyle measures were assessed at each wave and included Smoking status (current, former, never) and Alcohol consumption assessed using the Alcohol Use Disorders Identification Test (AUDIT^18^). For men, weekly alcohol consumption was categorized from 1 to 5 as abstainer, light (1–13 units), moderate (14–27 units), hazardous (28–42 units), or harmful (> 42 units) (i.e. 1 = abstainer; 5 = harmful). For women, weekly alcohol consumption was divided into abstainer, light (1–7 units), moderate (8–13 units), hazardous (14–28 units) or harmful (> 28 units) categories where a unit equates 10 g of alcohol. Physical activity was scored as the number of self-reported hours of performing activities at each of three intensity levels: mild, moderate and vigorous activities^12^, and these were coded as three separate variables. Cognitive engagement was a composite measure of life engagement^19^ based on the six domains (Realistic, Investigative, Artistic, Social, Enterprising and Conventional) of the RIASEC scales^19,20^. Social support was a composite measure of positive and negative social exchange with family and friends^21^.

### Medical domain

Depression and anxiety symptoms were measured with the Goldberg scale^22^. Systolic and diastolic blood pressure were computed over two measurements using an Omron M4 monitor (Hoofddorp, the Netherlands) after a rest of at least 5 min. Participants were classified as hypertensive if their mean diastolic or systolic blood pressure measures were higher than 90 and 140 mm Hg, respectively, or if they were currently taking antihypertensive medication. Self-reported medical conditions were obtained each wave for diabetes, epilepsy, arthritis, cancer, prior history of stroke/mini-stroke or TIA, history of serious head-injury with > 15 min loss of consciousness and use of any medications in the past month for lowering cholesterol. Body mass index (BMI) categories (obesity, overweight and normal weight) were based on self-reported weight at each wave and measured height at baseline [weight(kg)/height^2^ (m^2^)]*.*

### Memory

Immediate recall was assessed in the face-to-face assessment by a trained interviewer as part of a cognitive test battery. The first trial of the California Verbal Learning Test^23^ was used as the measure. A repeat trial following a filler task was also conducted and provided a measure of delayed recall. Delayed recall was not comparable at wave 4 in the 60 s cohort due to use of multiple learning trials.

### Statistical analysis

Our approach was exploratory given the lack of evidence on the specific research questions we aimed to address. To address Aim (a), we used linear regression models to estimate the sex difference in memory at each wave for each cohort, adjusting for age, race, English speaking background and education. We also used linear mixed models with subject specific random intercepts to model the change in memory between baseline (wave 1) and final wave (wave 4) for men and women within each cohort. Sex differences in the change in memory between wave 1 and wave 4 were estimated using an interaction term between sex and wave of data collection with two levels for wave. To address Aim (b), means or proportions of dementia risk factors for men and women were estimated. We then analysed sex differences in change in risk factors between wave 1 and wave 4 and evaluated whether this change was associated with sex differences in the change in memory during this period. This was achieved by inclusion of a three-way interaction in the linear mixed model with lower order interaction terms between sex, risk factor, and wave. To fully explore these sex and risk factor effects, we finally investigated associations between change in risk factors and change in memory for men and women separately. This allowed for specification of how the change in each risk factor accounted for change in sex differences in memory. For example, it is possible to obtain a significant sex-by-wave by risk factor interaction that is caused either by the risk factor having an effect in only one sex, or by having different levels of effect in each sex. Without conducting the sex specific analysis, it is not possible to distinguish these possibilities. Models were adjusted for age, race, non-English speaking background, years of education and data collection wave.

To address longitudinal missing data in cognitive measures and covariates, the above regression models were analysed using multiple imputed datasets. Specifically, we considered thirty imputed datasets following multivariate normal imputation models^24^ in which binary variables are rounded using adaptive rounding^25^. All analyses were conducted using Stata version 16.0 (StataCorp, College Station, Texas, USA).

### Consent to participate

Informed consent was obtained from all individual participants included in the study.

### Ethics approval

Approval was obtained from the Human Ethics Committee of The Australian National University. The procedures used in this study adhere to the tenets of the Declaration of Helsinki.

## Results

### Estimation of sex differences in memory function and change over time

Women performed better than men on immediate recall and delayed recall, in all cohorts and at each Wave (Online Appendix, Fig. A1 and Table A2). Longitudinal analyses showed that in the 20 s cohort, there were no sex differences in either immediate or delayed memory change. In the 40 s cohort women improved more over time than men on immediate recall (β = 0.25, 95% CI 0.03–0.45; Online Appendix, Table A3). In the 60 s cohort, rate of decline in immediate recall was faster in women than men (β = − 0.27, 95% CI − 0.48 to − 0.06; Online Appendix, Table A3).

### Dementia risk factors for men and women

**Figure 1:**
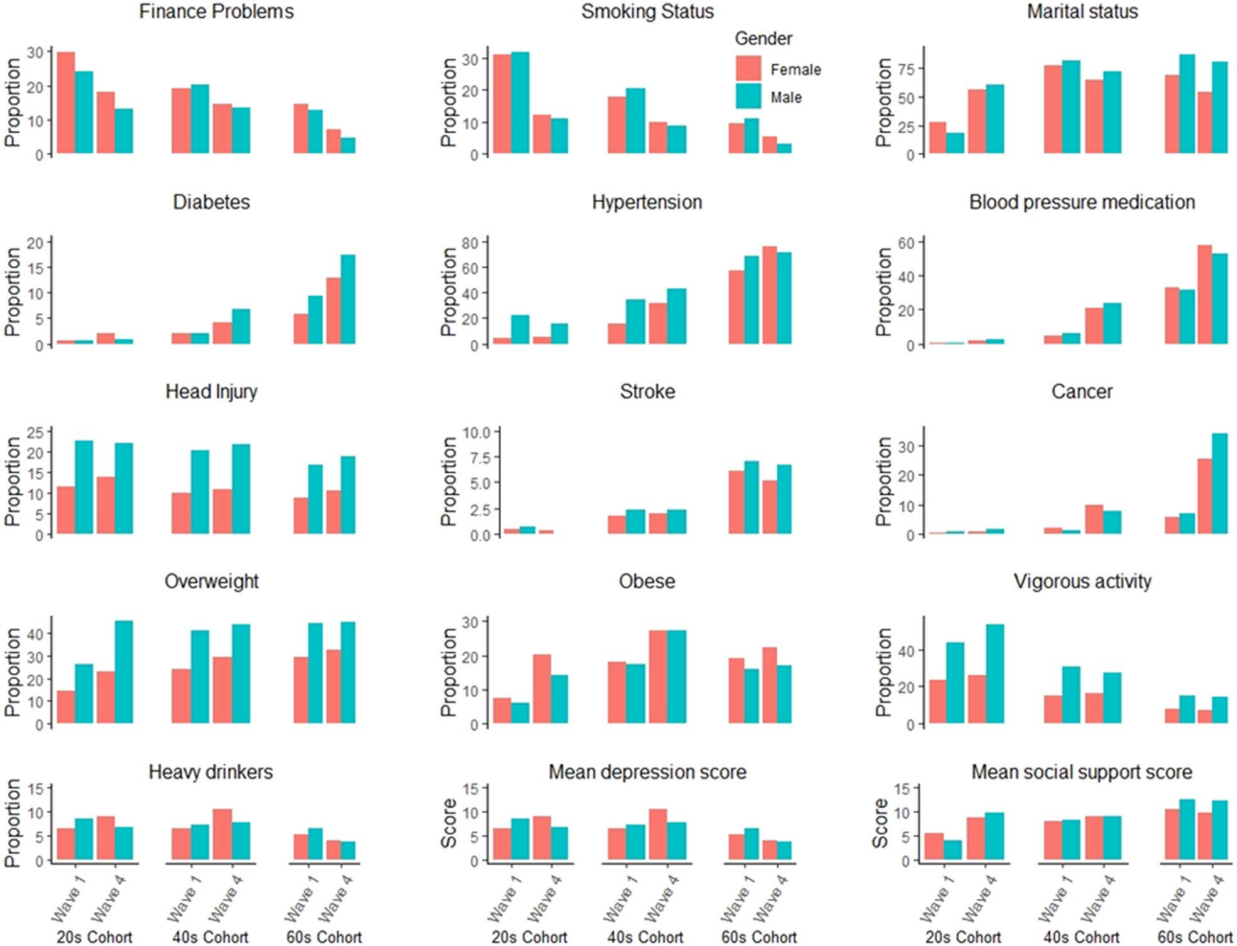
Prevalence of dementia risk factors at wave 1 and wave 4 in men and women in three cohorts.

Prevalence and average values for risk factor measures for men and women in each cohort at Wave 1 and Wave 4 are shown in Fig. 1 and in Table A4 of the Online Appendix. Notable sex differences were evident for baseline measures of head injury (22.8% in men vs 11.5% in women in the 20 s cohort), overweight (26% in men vs 14.6% in women in 20 s and 44.4% in men vs 29.4% in women in the 60 s), vigorous activity (43.3% in men versus 23.3% in women in the 20 s and 14.8% in men and 8% in women in the 60 s) and hypertension (higher in men until old age, i.e. 22% vs 3.9% in the 20 s; 34.8 versus 15.6% in 40 s and 67.9% vs 57.3% in the 60 s for men and women respectively at baseline). The proportion of men and women with hypertension increased with age but men consistently had higher rates than women. There was a notable increase in diabetes with age and wave, especially for men (0.5%, 2.0%, and 9.2% in men at baseline and 0.8%, 6.8% and 17.3% in men at wave 4 in the 20 s, 40 s and 60 s respectively). There was a notable increase in adults reporting taking blood pressure medication (0.5% and 0.2% in the 20 s at baseline to 52.5% and 57.9% in the 60 s at Wave 4 in men and women respectively), and a notable increase in rates of cancer with age (0.6 and 0.3% in the 20 s at baseline and 34.2% and 25.7% in the 60 s at Wave 4 for men and women respectively).

### Evaluation of whether sex differences in change in risk factors explains sex differences in change in memory

The linear mixed model analyses evaluating whether sex differences in change in dementia risk factors over time explained sex differences in late life memory ageing focussed on the 40 s and 60 s cohorts because of limited decline in memory in the 20 s cohort. Table 1 shows the risk factors significantly associated with change in memory in the 40 s cohort and their interaction with sex and wave (full results reported in the Online Appendix, Table A5). Current smoking (β = − 0.39, 95% CI − 0.69 to − 0.10, p = 0.01), moderate alcohol drinking (β = 0.46, 95% CI 0.19–0.73, p < 0.01), moderate physical activity (β = 0.41, 95% CI 0.11–0.71, p < 0.01), cognitive engagement (β = 0.58, 95% CI 0.17–0.99, p < 0.01), social support (β = 0.02, 95% CI 0.01–0.04, p < 0.01) and depression (β = − 0.06, 95% CI − 0.11 to − 0.01, p = 0.02) were associated with memory. Sex differences in social support (β = − 0.03, 95% CI − 0.05 to − 0.01, p = 0.02) had a significant interaction with memory in the 40 s cohort but no sex-by-time-by-risk factor interactions were significant.

**Table 1:** Dementia risk factors effects on memory performance, and dementia risk factor interactions with sex, and sex and wave (40 s cohort).

	Reg coef.	95% CI	p-value
**Genetic**			
APOE e4 (−/−)	Ref.		
APOE e4 (−/+)	0.20	− 0.08, 0.47	0.16
APOE e4 (+/+)	0.27	− 0.56, 1.10	0.52
Interaction (male × APOE e4 (−/−))	Ref.		
Interaction (female × APOE e4 (−/+))	− 0.18	− 0.55, 0.19	0.34
Interaction (female × APOE e4 (+/+))	− 1.06	− 2.16, − 0.04	0.06
**Demographic**			
Childhood financial hardship	− 0.32	− 0.66. 0.01	0.06
Interaction (sex × childhood financial hardship)	0.31	− 0.14, 1.20	0.18
Finance problem	− 0.26	− 0.54, 0.01	0.06
Interaction (sex × finance problem)	0.18	− 0.19, 0.55	0.35
Interaction (sex × finance problem × wave)	− 0.03	− 0.67, 0.61	0.93
**Lifestyle**			
Currently smoking	− **0.39**	− **0.69, **− **0.10**	**0.01**
Interaction (sex × currently smoking)	0.35	− 0.06, 0.77	0.09
Interaction (sex × currently smoking × wave)	− 0.39	− 1.11, 0.32	0.28
Moderate alcohol drinking	**0.46**	**0.19, 0.73**	**< 0.01**
Interaction (sex × moderate drinking)	− 0.33	− 0.67, 0.02	0.07
Interaction (sex × moderate drinking × wave)	0.37	− 0.14, 0.88	0.16
Moderate physical activity	**0.41**	**0.11, 0.71**	**0.01**
Interaction (sex × moderate activity)	− 0.28	− 0.68, 0.11	0.15
Interaction (sex × moderate activity × wave)	0.23	− 0.41, 0.86	0.48
Cognitive engagement	**0.58**	**0.17, 0.99**	**< 0.01**
Interaction (sex × cognitive engagement)	− 0.35	− 0.86, 0.16	0.17
Interaction (sex × cognitive engagement × wave)	0.14	− 0.67, 0.95	0.74
Schuster-social support score	**0.02**	**0.01, 0.04**	**< 0.01**
Interaction (sex × SS)	− **0.03**	− **0.05, **− **0.01**	**0.02**
Interaction (sex × SS × wave)	0.02	− 0.01, 0.05	0.16
**Medical**			
Depression (Goldberg scales)	− **0.06**	− **0.11, **− **0.01**	**0.02**
Interaction (sex × depression)	0.02	− 0.05, 0.08	0.67
Interaction (sex × depression × wave)	− 0.04	− 0.14, 0.06	0.46

Bold values indicate p<0.05.

We also estimated the association between risk factors and memory separately for men and for women (Table 2) in the 40 s cohort. In terms of the domains of risk, demographic and lifestyle domains had a greater number of effects for men than women, and genetic, medical domains had more effects for women than men. Specifically, childhood financial hardship (β = − 0.32, 95% CI − 0.64–0.00, p = 0.05), finance problems (β = − 0.27, 95% CI − 0.54 to − 0.02, p = 0.05), depression (β = − 0.06, 95% CI − 0.11 to − 0.01, p = 0.02) and current smoking (β = − 0.40 95% CI − 0.68 to − 0.11, p < 0.01) were associated with memory decline in men. Moderate alcohol drinking (β = 0.40, 95% CI 0.20–0.72, p < 0.01), moderate physical activity (β = 0.41, 95% CI 0.12–0.69, p < 0.01), cognitive engagement (β = 0.59, 95% CI 0.19–0.99, p < 0.01) and social support (β = 0.02, 95% CI 0.01–0.04, p < 0.1) were all associated with less memory decline in men. In women, *APOE e4*+/+ status (β = − 0.78, 95% CI − 1.56 to − 0.01, p = 0.05), stroke (β = − 0.092, 95% CI − 1.83 to − 0.01, p = 0.05) and hypertension (β = − 0.48, 95% CI 0.87 to − 0.09, p = 0.02) were associated with memory decline. The latter findings translate to incident hypertension being associated with a reduction of 0.48 of one item recalled on the memory test.

**Table 2:** Sex specific effects of risk factors on memory, and risk factor by wave interaction effects on memory decline (40 s cohort).

	Male			Female		
	Reg Coef.	95% CI	p-value	Reg Coef.	95% CI	p-value
**Genetic**						
APOE e4 (−/−)	Ref.					
APOE e4 (−/+)	0.20	− 0.06, 0.46	0.13	0.01	− 0.26, 0.28	0.95
APOE e4 (+/+)	0.26	− 0.54, 1.06	0.52	− **0.78**	− **1.56, **− **0.01**	**0.05**
**Demographic**						
Childhood financial hardship	− **0.32**	− **0.64, 0.00**	**0.05**	− 0.02	− 0.35, 0.30	0.89
Finance problem	− **0.27**	− **0.54, **− **0.02**	**0.05**	− 0.08	− 0.35, 0.19	0.55
Interaction (wave × finance problem)	− 0.11	− 0.58, 0.35	0.63	− 0.15	− 0.59, 0.30	0.52
**Lifestyle**						
Currently smoking	− **0.40**	− **0.68, **− **0.11**	**< 0.01**	− 0.04	− 0.34, 0.27	0.82
Interaction (wave × currently smoking)	− 0.02	− 0.53, 0.49	0.93	− 0.42	− 0.92, 0.08	0.10
Moderate drinking	**0.40**	**0.20, 0.72**	**< 0.01**	0.14	− 0.08, 0.39	0.25
Interaction (wave × moderate drinking)	− 0.21	− 0.58, 0.16	0.27	0.16	− 0.18, 0.49	0.37
Moderate activity	**0.41**	**0.12, 0.69**	**< 0.01**	0.13	− 0.13, 0.40	0.33
Interaction (wave × moderate activity)	− 0.02	− 0.48, 0.44	0.93	0.20	− 0.25, 0.66	0.38
Cognitive engagement	**0.59**	**0.19, 0.99**	**< 0.01**	0.23	− 0.09, 0.55	0.16
Interaction (wave × cognitive engagement)	− 0.05	− 0.66, 0.56	0.87	0.07	− 0.46, 0.60	0.79
Schuster-social support score	**0.02**	**0.01, 0.04**	**< 0.01**	− 0.001	− 0.02, 0.01	0.85
Interaction (wave × social support score)	− 0.01	− 0.03, 0.01	0.17	0.01	− 0.01, 0.03	0.53
**Medical**						
Hypertension	0.10	− 0.14, 0.34	0.40	0.30	− 0.01, 0.61	0.06
Interaction (wave × hypertension)	− 0.08	− 0.42, 0.26	0.65	− **0.48**	− **0.87, **− **0.09**	**0.02**
Stroke	− 0.05	− 0.82, 0.71	0.89	− **0.92**	− **1.83, **− **0.01**	**0.05**
Interaction (wave × stroke)	0.30	− 0.73, 1.33	0.57	− 0.53	− 1.58, 0.51	0.32
Depression (Goldberg scales)	− **0.06**	− **0.11, **− **0.01**	**0.02**	− 0.04	− 0.09, 0.00	0.07
Interaction (wave × depression)	0.02	− 0.05, 0.09	0.58	− 0.02	− 0.09, 0.05	0.61

Bold values indicate p<0.05.

**Figure 2:**
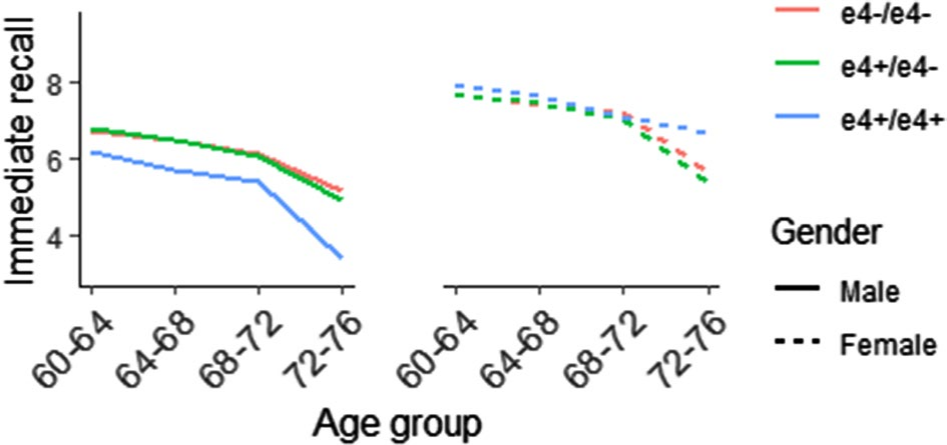
Raw scores for Immediate Recall over time for men and women by *APOE e4* genotype.

Table 3 shows the risk factors associated with memory decline in the 60 s (full results reported in the Online Appendix, Table A6). Main effects were significant for *APOE e4 (*++*)* (β = − 1.06, 95% CI − 1.78 to − 0.33, p = 0.01), finance problems (β = − 0.40, 95% CI − 0.71 to − 0.08, p = 0.02), moderate activity (β = 0.27 95% CI 0.02–0.52, p = 0.03), vigorous activity (β = 0.34, 95% CI 0.02–0.66, p = 0.03), cognitive engagement (β = 0.31, 95% CI − 0.00–0.62, p = 0.05) and social support (β = 0.02, 95% CI 0.00–0.03, p = 0.02). *APOE* genotype had a significant interaction with sex (Fig. 2, β = 0.96, 95% CI − 0.01–1.94, p = 0.05) such that women who were *APOE e4*+/+ showed less decline in memory in their 70 s than all other genotypes. In contrast, men who were *APOE e4*+/+ had the lowest level of memory performance and the greatest decline of all genotype categories, in their 70 s.

**Table 3:** Dementia risk factors effects on memory performance, and dementia risk factor interactions with sex, and sex and wave (60 s cohort).

	Reg Coef.	95% CI	p-value
**Genetic**			
APOE e4 (−/−)	Ref.		
APOE e4 (−/+)	− 0.12	− 0.34, 0.11	0.31
APOE e4 (+/+)	− **1.06**	− **1.78, **− **0.33**	**0.01**
Interaction (male × APOE e4 (− /− ))	Ref.		
Interaction (female × APOE e4 (− / +))	− 0.07	− 0.40, 0.25	0.66
Interaction (female × APOE e4 (+ / +))	**1.00**	**0.04, 1.96**	**0.04**
**Demographic**			
Finance problem	− **0.40**	− **0.71, **− **0.08**	**0.02**
Interaction (sex × finance problem)	0.24	− 0.20, 0.68	0.28
Interaction (sex × finance problem × wave)	0.03	− 0.81, 0.88	0.94
**Lifestyle**			
Moderate activity	**0.27**	**0.02, 0.52**	**0.03**
Interaction (sex × moderate activity)	0.06	− 0.30, 0.43	0.73
Interaction (sex × moderate activity × wave)	0.11	− 0.41, 0.63	0.68
Vigorous activity	**0.34**	**0.02, 0.66**	**0.03**
Interaction (sex × vigorous activity)	− 0.16	− 0.68, 0.36	0.54
Interaction (sex × vigorous activity × wave)	0.12	− 0.67, 0.91	0.77
Cognitive engagement	**0.31**	− **0.00, 0.62**	**0.05**
Interaction (sex × cognitive engagement)	0.05	− 0.36, 0.46	0.80
Interaction (sex × cognitive engagement × wave)	− 0.00	− 0.57, 0.57	0.99
Schuster-social support score (SS)	**0.02**	**0.00, 0.03**	**0.02**
Interaction (sex × SS)	− 0.01	− 0.03, 0.01	0.24
Interaction (sex × SS × wave)	0.00	− 0.03, 0.03	0.97
**Medical**			
Hypertension	0.06	− 0.17, 0.30	0.58
Interaction (sex × hypertension)	− **0.37**	− **0.70, **− **0.05**	**0.02**
Interaction (sex × hypertension × wave)	0.25	− 0.24, 0.73	0.31

Bold values indicate p<0.05. Adjusted for age, race, non-English speaking background, year of education and data collection wave.

Investigation of the effects of risk factors were also evaluated for men and women separately (Table 4). Among men, memory performance was negatively associated with *APOE* genotype (β = − 1.07, 95% CI − 1.77 to − 0.36, p < 0.01) and with more finance problems (β = − 0.41, 95% CI − 0.72 to − 0.11, p = 0.01) and positively associated with moderate (β = 0.29, 95% CI 0.05–0.53, p = 0.02) and vigorous activity (β = 0.36, 95% CI 0.05–0.67, p = 0.02), cognitive engagement (β = 0.36, 95% CI 0.05–0.67, p = 0.02) and social support (β = 0.02, 95% CI 0.00–0.03, p = 0.02). Among women, memory was negatively associated with alcohol abstention (β = − 0.46, 95% CI − 0.76 to − 0.17, p ≤ 0.01), hypertension (β = − 0.31, 95% CI − 0.54 to − 0.08, p = 0.01), and depression (β = − 0.09, 95% CI − 0.15 to − 0.03, p < 0.01) and positively associated with moderate activity (β = 0.33, 95% CI 0.06–0.60, p = 0.02) and cognitive engagement (β = 0.33, 95% CI 0.05–0.62, p = 0.02). Importantly, change in stroke status over time (incident stroke) for men was significantly associated with decline in memory (β = − 0.56, 95% CI − 1.12 to − 0.01, p = 0.05).

**Table 4:** Sex specific effects of risk factors on memory, and risk factor-by-wave interaction effects on memory decline (60 s cohort).

	Male			Female		
	Reg Coef.	95% CI	p-value	Reg Coef.	95% CI	p-value
**Genetic**						
APOE e4 (−/−)	Ref.			Ref.		
APOE e4 (−/+)	− 0.12	− 0.34, 0.10	0.28	− 0.19	− 0.45, 0.06	0.14
APOE e4 (+/+)	− **1.07**	− **1.77, **− **0.36**	** < 0.01**	− 0.07	− 0.77,0.68	0.85
**Demographic**						
Finance problem	− **0.41**	− **0.72, **− **0.11**	**0.01**	− 0.14	− 0.47, 0.18	0.40
Interaction (wave × finance prob)	− 0.02	− 0.65, 0.61	0.94	− 0.01	− 0.59, 0.57	0.91
**Lifestyle**						
Alcohol abstention	− 0.11	− 0.42, 0.30	0.74	− **0.46**	− **0.76, **− **0.17**	** < 0.01**
Interaction (wave × alcohol abst)	− 0.12	− 0.58, 0.34	0.60	0.29	− 0.14, 0.72	0.18
Moderate physical activity	**0.29**	**0.05, 0.53**	**0.02**	**0.33**	**0.06, 0.60**	**0.02**
Interaction (wave × mod activity)	− 0.11	− 0.47, 0.25	0.55	0.00	− 0.39, 0.40	0.98
Vigorous physical activity	**0.36**	**0.05, 0.67**	**0.02**	0.16	− 0.27, 0.60	0.47
Interaction (wave × vig activity)	− 0.22	− 0.68, 0.25	0.36	− 0.09	− 0.871, 0.53	0.79
Cognitive engagement	**0.36**	**0.05, 0.67**	**0.02**	**0.33**	**0.05, 0.62**	**0.02**
Interaction (wave × cog engage)	− 0.07	− 0.48, 0.34	0.74	− 0.06	− 0.46, 0.34	0.77
Schuster-social support score	**0.02**	**0.00, 0.03**	**0.02**	0.01	− 0.01, 0.02	0.48
Interaction (wave × social support)	0.00	− 0.02, 0.02	0.90	0.00	− 0.02, 0.03	0.88
**Medical**						
Hypertension	0.06	− 0.16, 0.28	0.61	− **0.31**	− **0.54, **− **0.08**	**0.01**
Interaction (wave × hypertension)	− 0.02	− 0.31, 0.34	0.92	0.27	− 0.09, 0.63	0.14
Stoke	0.04	− 0.37, 0.44	0.87	− 0.29	− 0.78, 0.19	0.24
Interaction (stroke × wave)	− **0.56**	− **1.12, **− **0.01**	**0.05**	− 0.19	− 0.83, 0.45	0.56
Depression	− 0.04	− 0.10, 0.01	0.13	− **0.09**	− **0.15, **− **0.03**	** < 0.01**
Interaction (wave × depression)	− 0.04	− 0.12, 0.03	0.26	− 0.01	− 0.08, 0.09	0.89

Bold values indicate p<0.05.

## Discussion

In the oldest cohort, women had greater decline in memory than men, despite their having a higher level of performance^26^. This is particularly interesting as episodic memory declines in prodromal AD and women have higher rates of AD^27–30^. For midlife women and late-life men, *APOE e4* was associated with faster decline in episodic memory. Overall, there were many sex differences in level, prevalence and incidence of risk factors drawn from multiple domains. In some cases, the size of sex differences in prevalence of risk and protective factors was large. For example, midlife men were twice as likely to participate in vigorous physical activity than midlife women, and about 50% more likely to have diabetes. In contrast, the sex differences in memory performance were relatively small and stable over time, except in later life when women showed more decline. In the 40 s cohort, of the seven factors significantly associated with episodic memory in men, four were protective (moderate drinking, moderate physical activity, cognitive engagement and social support). In contrast, only three factors were associated with memory among women in the 40 s cohort and none of these were protective. In addition, two factors affecting memory in women at mid-life had very limited modifiability (*APOE e4,* stroke). Surprisingly, we did not find an association between BMI category in mid-life and memory decline which differs from the literature on BMI and risk of dementia^31,32^. In the oldest cohort, of the seven factors significant for men, four were protective (moderate activity, vigorous activity, cognitive engagement, social support), and of the five factors affecting women, only two were protective (moderate activity and cognitive engagement). The only risk factor that explained sex differences over time was stroke which increased the risk of memory decline in men but not in women. Hypertension was negatively associated with memory performance among women (but not men) in both cohorts.

Overall, the pattern of sex differences in risk factor effects leads us to speculate that the higher rates of dementia in women in late-life may be due, at least in part, to their having fewer modifiable risk factors over the life-course, particularly in middle age but greater susceptibility to the effect of hypertension. Some prior studies suggest midlife hypertension predicts dementia in women, despite higher prevalence of midlife hypertension in men^33,34^. However, in the Victoria Longitudinal Study (VLS) we previously reported that memory resilience (defined as high and stable memory test performance) amongst *APOE e4* carriers was associated with more modifiable factors in women than men aged 53 to 95 years^35^. Our present study identified similar factors were protective against memory decline in older women (cognitive and moderate physical activity) as were associated with memory resilience in the VLS, but our analyses did not stratify by *APOE* genotype and these differences in design limit direct comparison. It is possible that risk factor effects may differ in the age at which they begin to impact dementia risk. This would mean that even if the same factors increase the risk of dementia for men and women, they may commence their impact at different ages leading to different trajectories of memory decline. Overall longevity differences between men and women may also influence observed sex differences in patterns of risk.

Our findings in relation to *APOE* are somewhat consistent with the results of a recent systematic review that examined the risk of AD according to age and sex^36^ and *APOE* genotype. That review of 58,000 adults aged 55 to 85 also found that *APOE e4* impacted women’s memory performance in middle age but not later life. Our findings also suggest that women are more vulnerable to the effects of midlife hypertension on memory, than men. If this is a cumulative effect, then duration of hypertension may be a critical factor that differs between men and women. Sex differences in the effect of blood pressure on brain structure was found in a subsample of the older cohort who had longitudinal neuroimaging data such that higher mean blood pressure was associated with better cognition and larger brain volumes in men, but with reduced brain volumes and poorer cognition in women^37^. In addition to direct biological relationships between blood pressure and cognition, there may be sex differences in the detection and treatment of hypertension that contribute to the finding that blood pressure impacts cognitive function of men and women differently^38^.

Limitations of our study are the lack of neuropathological and genetic biomarkers beyond *APOE*, self-reported medical conditions, and small cell sizes for some three-way comparisons. Many protective factors may be impacted by incipient cognitive difficulties in late-life and so results in longitudinal studies may be subject to reverse causation. The sample was drawn from a relatively well educated Australian urban population and was largely Caucasian which may limit generalisability. Our analysis approach only used two waves of data which was required to address our specific research question. Alternative methods using trajectory analyses include all available data-points^39^ but do not specifically address the questions addressed in this study regarding differences in differences (i.e. differences in risk factors explaining differences in memory change). Specific questions about the nature of sex differences require carefully specified statistical models.

Strengths of our study include the evaluation of memory using the same measures in three cohorts over a 12-year follow-up period. The cohorts were also population-based and we evaluated a large number of risk factors. Incorporation of data from across the adult life-course enabled clarification that the sex differences in memory present in old age do not emerge in late-life, but are relatively constant from young adulthood.

We conclude that risk factors for memory decline in ageing draw from multiple domains and differentially across the life course, an observation that is consistent with earlier life course theories of the aetiology of dementia^40^. Relative to the sex differences in memory, the sex differences in dementia risk factors are larger. Men had more modifiable dementia risk factors than women. It is possible that a large number of small effects of sex differences may contribute to the overall sex differences observed. The current study provides insights into sex differences in dementia risk but further work is needed to evaluate the relationship of these risk factors to a wider range of chronic diseases. A broader understanding of overall patterns of risk factors for cardiometabolic disease and neurodegeneration is needed in order to inform tailored (potentially sex specific) interventions. The key risk factors identified in this study that were sex specific and explained sex differences in memory ageing were midlife hypertension (increased risk in women compared to men) and stroke (increased risk in older men). These factors are interrelated, with hypertension being the major risk factor for stroke. Our findings provide further support for the use of multidomain risk reduction approaches to promote cognitive health and increased awareness of hypertension in midlife women and stroke prevention in men.

## Supplementary Information

Supplementary Information.

## Data Availability

The authors confirm that the data supporting the findings of this study are available within the article and its supplementary materials.
