# Supplementary material for: Association of sex differences in dementia risk factors with sex differences in memory decline in a population-based cohort spanning 20–76 years
Source: Sci Rep. 2021 Apr 8;11:7710. doi: 10.1038/s41598-021-86397-7 (PMC8032756; doi:10.1038/s41598-021-86397-7)
Supplement: Supplementary file 1 — Supplementary Information. [file 41598_2021_86397_MOESM1_ESM.docx]

**Supplementary Tables and Figures**

TITLE

Association of Sex differences in dementia risk factors with Sex differences in memory decline in three population-based cohorts spanning 20-76 years

JOURNAL NAME:

Scientific Reports

AUTHORS:

Kaarin J. Anstey^1,2,3^, Ruth Peters^1,2^, Moyra Mortby^1,2^, Kim M. Kiely^1,2^, Ranmalee Eramudugolla^1,2^ Nicolas Cherbuin^3^, Hamidul Huque^1^, Roger A. Dixon^4,5^

AFFILIATIONS:

^1^School of Psychology, University of New South Wales, Sydney, Australia

^2^Neuroscience Research Australia, Sydney, Australia

^3^Centre for Research on Ageing Health and Wellbeing, School of Population Health, The Australian National University, Canberra, Australia

^4^Department of Psychology, University of Alberta, Edmonton, Canada

^5^Neuroscience and Mental Health Institute, University of Alberta, Edmonton, Canada

CORRESPONDING AUTHOR: Kaarin J. Anstey

Postal address: Neuroscience Research Australia, 139 Barker Street, Randwick, NSW 2031, Australia

Email address: [k.anstey@unsw.edu.au](mailto:k.anstey@unsw.edu.au)


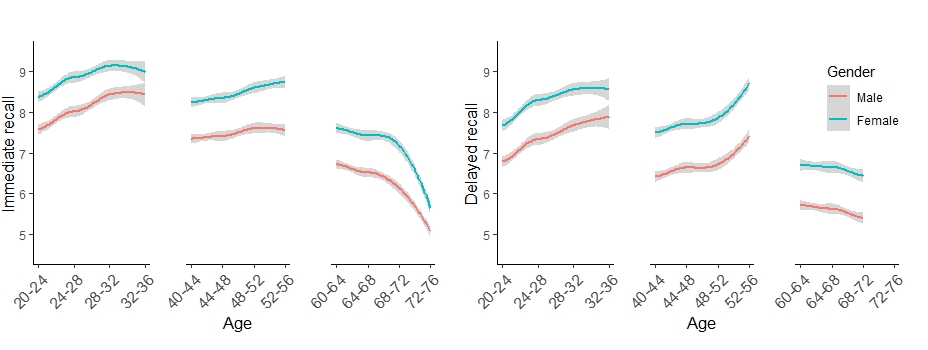


**Fig A1** Trajectories of immediate and delayed recall by cohort and sex over 4 waves. Grey shading represents error range.

**Table A1**: Sample sizes at each wave and baseline characteristics of PATH cohorts

| Variables | 20s cohort | 40s cohort | 60s cohort |
| --- | --- | --- | --- |
| **Sample sizes (n)** |  |  |  |
| Wave 1 | 2404 (100.0) | 2530 (100.0) | 2509 (100.0) |
| Wave 2 | 2139 (89.0) | 2354 (93.0) | 2197 (87.3) |
| Wave 3 | 1978 (82.3) | 2182 (86.2) | 1954 (77.9) |
| Wave 4 | 1191 (49.5) | 1774 (70.1) | 1632 (65.0) |
| **Age (Mean ± SD)** | 22.6±1.5 | 42.6±1.5 | 62.5±1.5 |
| **Year of education (Mean ± SD)** | 14.6±1.6 | 14.6±2.3 | 13.9±2.8 |
| **Male Sex** | 1162 (48.3) | 1193 (47.2) | 1289 (51.4) |
| **Race** |  |  |  |
| Caucasian | 2196 (91.4) | 2386 (94.3) | 2403 (95.9) |
| Asian | 82 (3.4) | 88 (3.5) | 62 (2.5) |
| Other | 106 (4.4) | 54 (2.1) | 41 (1.6) |
| Missing | 20 (0.8) | 2 (0.1) | 3 (0.1) |
| **Non-English speaking (Yes)** | 30 (1.3) | 81 (3.2) | 240 (9.6) |
| ***APOE4*** |  |  |  |
| Homozygous (-‘ve) | 1673 (69.6) | 1661 (65.7) | 1712 (68.2) |
| Heterozygous | 558 (23.2) | 591 (23.4) | 586 (23.4) |
| Homozygous (+’ve) | 60 (2.5) | 46 (1.8) | 49 (2) |
| Missing | 113 (4.7) | 232 (9.2) | 162 (6.5) |
| **Childhood poverty or financial hardship (Yes)** | 160 (6.7) | 306 (12.1) | 377 (15.0) |
| **Attrition** |  |  |  |
| Died on or before wave 4 | 4 (0.2) | 40 (1.6) | 352 (14.0) |
| Lost to follow-up | 663 (27.6) | 716 (28.3) | 525 (25.9) |

**Table A2**: Average sex difference in cognitive measures after adjusting for age, race, non-English speaking background, years of education in the 20s, 40s and 60s age groups

|  | Immediate recall | | | | | Delayed recall | | | | | |
| --- | --- | --- | --- | --- | --- | --- | --- | --- | --- | --- | --- |
|  | Male | Female | | Adjusted Diff (95% CI) | | Male | | Female | Adjusted Difference (95% CI) | | |
| 20s |  |  | |  | |  | |  |  | | |
| Wave 1 - 20 to 24 years | 7.58 | 8.38 | 0.75 (0.58, 0.91) | | 6.78 | | 7.67 | | | 0.83 (0.65, 1.01) |  |
| Wave 2 - 24 to 28 years | 8.02 | 8.86 | | 0.78 (0.58, 0.97) | | 7.34 | | 8.30 | 0.89 (0.67, 1.10) | | |
| Wave 3 -28 to 32 years | 8.44 | 9.14 | | 0.65 (0.45, 0.85) | | 7.68 | | 8.56 | 0.81 (0.59, 1.03) | | |
| Wave 4 - 32 to 36 years | 8.44 | 8.99 | | 0.52 (0.13, 0.92) | | 7.89 | | 8.56 | 0.67 (0.25, 1.09) | | |
| 40s |  |  | |  | |  | |  |  | | |
| Wave 1 - 40 to 44 years | 7.34 | 8.24 | | 0.98 (0.81, 1.15) | | 6.41 | | 7.50 | 1.18 (0.99, 1.36) | | |
| Wave 2 - 44 to 48 years | 7.42 | 8.35 | | 1.01 (0.83, 1.19) | | 6.64 | | 7.71 | 1.14 (0.94, 1.33) | | |
| Wave 3 - 48 to 52 years | 7.61 | 8.60 | | 1.07 (0.89, 1.25) | | 6.72 | | 7.86 | 1.22 (1.02, 1.42) | | |
| Wave 4 - 52 to 56 years | 7.55 | 8.75 | | 1.27 (1.05, 1.49) | | 7.43 | | 8.73 | 1.37 (1.14, 1.59) | | |
| 60s |  |  | |  | |  | |  |  | | |
| Wave 1 - 60 to 64 years | 6.72 | 7.61 | | 1.03 (0.86, 1.21) | | 5.71 | | 6.69 | 0.02 (0.01, 0.02) | | |
| Wave 2 - 64 to 68 years | 6.51 | 7.43 | | 1.09 (0.91, 1.27) | | 5.62 | | 6.65 | 0.02 (0.01, 0.02) | | |
| Wave 3 - 68 to 72 years | 6.13 | 7.17 | | 1.23 (1.03, 1.43) | | 5.39 | | 6.43 | 0.02 (0.01, 0.02) | | |
| Wave 4 - 72 to 76 years | 5.05 | 5.63 | | 0.73 (0.55, 0.92) | | NA | | NA |  | | |

*Delayed recall at wave 4 are not comparable with that of earlier waves.

**Table A3**: Multilevel Model estimates (and 95% confidence interval) the change in episodic memory between baseline and final wave with respect to participant sex.

|  | **20s** | | **40s** | | **60s** | |
| --- | --- | --- | --- | --- | --- | --- |
|  | Immediate Recall | Delayed Recall | Immediate Recall | Delayed Recall | Immediate Recall | Delayed Recall* |
| Fixed effects |  |  |  |  |  |  |
| **Age** | -0.06 (-0.11, -0.00) | -0.04 (-0.10, 0.02) | 0.00 (-0.05, 0.05) | -0.01 (-0.06, 0.05) | -0.07 (-0.12, -0.03) | -0.05 (-0.10, 0.00) |
| **Female Sex** | 0.74 (0.57, 0.91) | 0.83 (0.64, 1.02) | 0.99 (0.82, 1.16) | 1.18 (0.99, 1.36) | 1.03 (0.87, 1.19) | 1.13 (0.95, 1.32) |
| **Race** |  |  |  |  |  |  |
| Caucasian | Ref | Ref | Ref | Ref | Ref | Ref |
| Asian | -0.74 (-1.21, -0.27) | -0.74 (-1.26, -0.23) | -1.17 (-1.64 -0.71) | -1.24 (-1.74, -0.74) | -0.45 (-0.93, 0.04) | -0.47 (-1.04, 0.11) |
| Other | -0.81 (-1.22, -0.41) | -0.75 (-1.19, -0.31) | -1.35 (-1.89, -0.82) | -1.00 (-1.58, -0.41) | -0.50 (-1.06, 0.06) | -0.66 (-1.31, -0.00) |
| **Non-English speaking** | -0.30 (-1.05, 0.45) | -0.59 (-1.41, 0.23) | -0.58 (-1.06, -0.10) | -0.98 (-1.50, -0.46) | -0.82 (-1.09, -0.56) | -0.77 (-1.08, -0.47) |
| **Year of education** | 0.25 (0.20, 0.30) | 0.24 (0.19, 0.30) | 0.19 (0.16, 0.23) | 0.20 (0.16, 0.23) | 0.19 (0.17, 0.22) | 0.18 (0.15, 0.21) |
| **Final wave vs wave 1** | 1.07 (0.36, 1.78) | 1.14 (0.37, 1.91) | 0.02 (-0.62, 0.65) | 0.90 (0.22, 1.59) | -0.92 (-1.51, -0.34) | -0.05 (-0.49, 0.40) |
| **Interaction (Wave* Sex)** | -0.07 (-0.43, 0.29) | -0.03 (-0.41, 0.35) | 0.25 (0.03, 0.47) | 0.18 (-0.05, 0.41) | -0.27 (-0.48, -0.06) | 0.09 (-0.10, 0.29) |
| **Variance components** |  |  |  |  |  |  |
| Var (Intercept) | 1.54 | 2.16 | 1.39 | 1.52 | 1.14 | 1.61 |

*Delayed recall for 60s cohorts were compared between wave 1 and wave 3

**Table A4:** Level of various demographic, lifestyle, medical, pharmacological and environmental risk factors among men and women at baseline and wave 4 in each cohort

|  | 20s | | | | 40s | | | | 60s | | | | |
| --- | --- | --- | --- | --- | --- | --- | --- | --- | --- | --- | --- | --- | --- |
|  | Wave 1 | | Wave 4 | | Wave 1 | | Wave 4 | | Wave 1 | | Wave 4 | | |
|  | Male n=1162 | Female n=1242 | Male n=496 | Female n=695 | Male n=1193 | Female n=1337 | Male n=822 | Female n=952 | Male n=1289 | Female n=1220 | Male n=846 | Female n=786 |  |
| **Biomarker** |  |  |  |  |  |  |  |  |  |  |  |  |  |
| APOE e4 (-/-) | 68.2 | 70.9 | NA | NA | 65.1 | 66.2 | NA | NA | 68.1 | 68.4 | NA | NA |  |
| APOE e4 (-/+) | 22.4 | 20.3 | NA | NA | 22.0 | 20.2 | NA | NA | 21.6 | 20.3 | NA | NA |  |
| APOE e4 (+/+) | 2.3 | 1.9 | NA | NA | 1.7 | 1.9 | NA | NA | 1.8 | 2.1 | NA | NA |  |
| **Demographic** |  |  |  |  |  |  |  |  |  |  |  |  |  |
| Childhood poverty | 5.9 | 7.3 | NA | NA | 11.8 | 12.3 | NA | NA | 17.2 | 12.7 | NA | NA |  |
| Finance Problem | 24.2 | 30.0 | NA | NA | 20.2 | 19.1 | NA | NA | 12.8 | 14.8 | NA | NA |  |
| Married/Partnered | 18.5 | 27.6 | 60.3 | 56.0 | 81.4 | 77.4 | 72.5 | 64.8 | 86.8 | 68.8 | 80.8 | 54.4 |  |
| **Lifestyle** |  |  |  |  |  |  |  |  |  |  |  |  |  |
| Currently smoking | 31.7 | 30.8 | 10.9 | 12.1 | 20.4 | 17.8 | 8.9 | 10.0 | 11.4 | 9.6 | 3.1 | 5.2 |  |
| Moderate drinking | 83.6 | 83.0 | 88.7 | 79.6 | 84.8 | 82.2 | 82.4 | 79.4 | 83.9 | 76.0 | 86.3 | 77.5 |  |
| Alcohol abstains | 7.2 | 9.3 | 4.6 | 11.4 | 7.6 | 10.9 | 9.4 | 9.7 | 9.4 | 18.4 | 9.6 | 17.9 |  |
| Heavy drinking | 8.5 | 6.5 | 6.6 | 8.9 | 7.3 | 6.4 | 7.8 | 10.5 | 6.4 | 5.3 | 3.7 | 4.1 |  |
| Mild activity | 31.2 | 49.0 | 16.9 | 32.8 | 26.3 | 43.2 | 21.2 | 31.0 | 40.1 | 55.0 | 29.8 | 42.5 |  |
| Moderate activity | 20.7 | 22.8 | 25.6 | 33.1 | 37.1 | 33.3 | 31.9 | 27.4 | 32.7 | 25.3 | 39.4 | 32.6 |  |
| Vigorous activity | 43.3 | 23.3 | 53.2 | 26.3 | 30.8 | 15.2 | 27.6 | 16.1 | 14.8 | 8.0 | 14.2 | 7.6 |  |
| Cognitive engagement | 90.0 | 86.6 | 96.6 | 91.5 | 90.1 | 84.5 | 91.6 | 89.4 | 84.9 | 78.9 | 85.1 | 76.0 |  |
| Sleep problem | 41.0 | 51.0 | 51.4 | 57.8 | 43.5 | 44.9 | 47.6 | 59.6 | 29.2 | 36.1 | 32.7 | 44.0 |  |
| Overweight | 26.0 | 14.6 | 45.0 | 22.7 | 41.1 | 24.1 | 43.8 | 29.3 | 44.4 | 29.4 | 44.8 | 32.6 |  |
| Obese | 6.0 | 7.6 | 14.3 | 20.4 | 17.3 | 18.1 | 27.5 | 27.3 | 15.9 | 19.2 | 16.9 | 22.5 |  |
| Schuster-social support score | 4.1±5.9 | 5.4±6.5 | 9.6±7.6 | 8.8±7.9 | 8.3±7.8 | 7.9±8.2 | 9.0±8.4 | 8.9±8.5 | 12.4±7.5 | 10.4±7.8 | 12.1±6.7 | 9.8±6.9 |  |
| **Medical** |  |  |  |  |  |  |  |  |  |  |  |  |  |
| Arthritis | 1.7 | 2.7 | 5.0 | 7.0 | 9.9 | 10.9 | 24.2 | 38.2 | 25.8 | 45.4 | 51.4 | 69.6 |  |
| Cancer | 0.6 | 0.3 | 1.8 | 0.7 | 1.3 | 2.2 | 7.8 | 9.7 | 7.0 | 5.7 | 34.2 | 25.7 |  |
| Anxiety (Goldberg scales) | 3.2±2.6 | 4.4±2.7 | 3.3±2.6 | 4.2±2.7 | 3.3±2.7 | 3.7±2.7 | 2.9±2.5 | 3.5±2.6 | 2.0±2.2 | 2.5±2.4 | 2.0±2.0 | 2.4±2.2 |  |
| Depression (Goldberg scales) | 2.5±2.3 | 3.2±2.4 | 2.2±2.3 | 2.8±2.3 | 2.3±2.3 | 2.6±2.4 | 2.1±2.3 | 2.2±2.3 | 1.5±1.8 | 1.8±1.9 | 1.5±1.7 | 1.7±1.8 |  |
| Diabetes (Yes) | 0.5 | 0.5 | 0.8 | 2.0 | 2.0 | 2.0 | 6.8 | 4.2 | 9.2 | 5.8 | 17.3 | 12.8 |  |
| Hearing loss | NA | NA | NA | NA | NA | NA | NA | NA | 0.1 | 0.1 | 17.7 | 7.0 |  |
| Hypertension (Yes) | 22.0 | 3.9 | 15.9 | 5.5 | 34.8 | 15.6 | 43.3 | 31.6 | 67.9 | 57.3 | 71.4 | 75.6 |  |
| Stroke (Yes) | 0.7 | 0.5 | 0.0 | 0.4 | 2.4 | 1.8 | 2.4 | 2.0 | 7.1 | 6.1 | 6.7 | 5.2 |  |
| Head injury (yes) | 22.8 | 11.5 | 22.0 | 13.7 | 20.4 | 9.9 | 21.8 | 10.9 | 16.7 | 8.8 | 18.9 | 10.6 |  |
| **Pharmacological** |  |  |  |  |  |  |  |  |  |  |  |  |  |
| Cholesterol medication (Yes) | NA | NA | NA | NA | NA | NA | NA | NA | 25.4 | 20.4 | 47.3 | 41.1 |  |
| Anti-hypertensive (Yes) | 0.5 | 0.2 | 2.6 | 1.7 | 5.9 | 4.7 | 23.4 | 21.1 | 31.8 | 33.1 | 52.5 | 57.9 |  |

**Table A5:** Analysis of association between individual risk factors and change in immediate recall between wave 1 and wave 4 for the 40s cohort

|  | Reg Coef | 95% CI | p-value |
| --- | --- | --- | --- |
| **Biomarker** |  |  |  |
| APOE e4 (-/-) | Ref |  |  |
| APOE e4 (-/+) | 0.20 | -0.08, 0.47 | 0.16 |
| APOE e4 (+/+) | 0.27 | -0.56, 1.10 | 0.52 |
| Interaction (Male* APOE e4 (-/-)) | Ref |  |  |
| Interaction (Female* APOE e4 (-/+)) | -0.18 | -0.54, 0.19 | 0.34 |
| Interaction (Female*APOE e4 (+/+)) | -1.06 | -2.16, 0.04 | 0.06 |
| **Demographic** |  |  |  |
| Childhood financial hardship | -0.32 | -0.66. 0.01 | 0.06 |
| Interaction (Sex* Childhood financial hardship) | 0.31 | -0.14, 1.20 | 0.18 |
| Finance problem | -0.26 | -0.54, 0.01 | 0.06 |
| Interaction (Sex* Finance problem) | 0.18 | -0.19, 0.55 | 0.35 |
| Interaction (Sex* Finance problem* wave) | -0.03 | -0.67, 0.61 | 0.93 |
| Partnership status | -0.25 | -0.55, 0.05 | 0.10 |
| Interaction (Sex* Partnership status**)** | 0.04 | -0.36, 0.44 | 0.84 |
| Interaction (Sex* Partnership status*wave**)** | -0.04 | -0.60, 0.48 | 0.87 |
| **Lifestyle** |  |  |  |
| Currently smoking | **-0.39** | **-0.69, -0.10** | **0.01** |
| Interaction (Sex* Currently smoking**)** | 0.35 | -0.06, 0.77 | 0.09 |
| Interaction (Sex* Currently smoking*wave**)** | -0.39 | -1.11, 0.32 | 0.28 |
| Alcohol abstains | -0.33 | -0.77, 0.12 | 0.15 |
| Interaction (Sex* Alcohol abstains**)** | 0.15 | -0.43, 0.71 | 0.63 |
| Interaction (Sex* Alcohol abstains *wave**)** | 0.28 | -0.52, 1.08 | 0.52 |
| Moderate drinking | **0.46** | **0.19, 0.73** | **<0.01** |
| Interaction (Sex* Moderate drinking**)** | -0.33 | -0.67, 0.02 | 0.07 |
| Interaction (Sex* Moderate drinking*wave**)** | 0.37 | -0.14, 0.88 | 0.16 |
| Moderate activity | **0.41** | **0.11, 0.71** | **0.01** |
| Interaction (Sex* Moderate activity**)** | -0.28 | -0.68, 0.11 | 0.15 |
| Interaction (Sex* Moderate activity*wave**)** | 0.23 | -0.41, 0.86 | 0.48 |
| Vigorous activity | 0.13 | -0.18, 0.44 | 0.37 |
| Interaction (Sex* Vigorous activity**)** | -0.29 | -0.75, 0.16 | 0.20 |
| Interaction (Sex* Vigorous activity*wave**)** | 0.24 | -0.44, 0.92 | 0.48 |
| Cognitive engagement | **0.58** | **0.17, 0.99** | **<0.01** |
| Interaction (Sex* Cognitive engagement**)** | -0.35 | -0.86, 0.16 | 0.17 |
| Interaction (Sex* Cognitive engagement *wave**)** | 0.14 | -0.67, 0.95 | 0.74 |
| Sleep problem | -0.07 | -0.31, 0.16 | 0.54 |
| Interaction (Sex*Sleep problem**)** | -0.01 | -0.33, 0.31 | 0.96 |
| Interaction (Sex*Sleep problem *wave**)** | 0.11 | -0.36, 0.58 | 0.65 |
| Overweight | -0.09 | -0.36, 0.18 | 0.50 |
| Interaction (Sex* Overweight**)** | 0.16 | -0.22, 0.54 | 0.40 |
| Interaction (Sex* Overweight *wave**)** | -0.28 | -0.84, 0.28 | 0.33 |
| Obese | 0.09 | -0.26, 0.43 | 0.62 |
| Interaction (Sex* Obese**)** | 0.10 | -0.35, 0.56 | 0.66 |
| Interaction (Sex* Obese *wave**)** | -0.07 | -0.67, 0.53 | 0.82 |
| Schuster-social support score | **0.02** | **0.01, 0.04** | **<0.01** |
| Interaction (Sex* SS**)** | **-0.03** | **-0.05, -0.01** | **0.02** |
| Interaction (Sex* SS*wave**)** | 0.02 | -0.01, 0.05 | 0.16 |
| **Medical** |  |  |  |
| Arthritis | 0.10 | -0.30, 0.49 | 0.63 |
| Interaction (Sex* Arthritis**)** | -0.30 | -0.83, 0.22 | 0.25 |
| Interaction (Sex* Arthritis *wave**)** | 0.45 | -0.19, 1.109 | 0.17 |
| Cancer | 0.25 | -0.76, 1.26 | 0.63 |
| Interaction (Sex* Cancer**)** | -0.40 | -1.66, 0.86 | 0.53 |
| Interaction (Sex* Cancer *wave**)** | 0.36 | -1.06, 1.75 | 0.63 |
| Diabetes | -0.40 | -1.23, 0.44 | 0.35 |
| Interaction (Sex* Diabetes**)** | 0.54 | -0.61, 1.70 | 0.36 |
| Interaction (Sex* Diabetes *wave**)** | -0.83 | -2.17, 0.51 | 0.23 |
| Hypertension | 0.10 | -0.15, 0.34 | 0.44 |
| Interaction (Sex* Hypertension**)** | 0.21 | -0.18, 0.60 | 0.29 |
| Interaction (Sex*Hypertension*wave**)** | -0.40 | -0.91, 0.11 | 0.12 |
| Stroke | -0.06 | -0.85, 0.73 | 0.87 |
| Interaction (Sex*Stroke**)** | -0.85 | -2.02, 0.34 | 0.16 |
| Interaction (Sex*Stroke*wave**)** | -0.84 | -2.31, 0.63 | 0.26 |
| Any head injury | -0.09 | -0.39, 0.22 | 0.58 |
| Interaction (Sex* Any head injury**)** | -0.13 | -0.62, 0.36 | 0.60 |
| Interaction (Sex* Any head injury *wave**)** | 0.02 | -0.59, 0.63 | 0.95 |
| Anxiety (Goldberg scales) | -0.03 | -0.08, 0.01 | 0.15 |
| Interaction (Sex* Anxiety**)** | 0.01 | -0.05, 0.07 | 0.81 |
| Interaction (Sex* Anxiety *wave**)** | -0.03 | -0.09, 0.08 | 0.93 |
| Depression (Goldberg scales) | **-0.06** | **-0.11, -0.01** | **0.02** |
| Interaction (Sex* Depression**)** | 0.02 | -0.05, 0.08 | 0.67 |
| Interaction (Sex* Depression *wave**)** | -0.04 | -0.14, 0.06 | 0.46 |
| **Pharmacological** |  |  |  |
| Blood pressure medication | 0.38 | -0.11, 0.87 | 0.13 |
| Interaction (Sex* BP medication**)** | -0.32 | -1.04, 0.39 | 0.37 |
| Interaction (Sex* BP medication *wave**)** | 0.02 | -0.78, 0.82 | 0.96 |

*Adjusted for age, race, non-English speaking background, year of education, wave. Reference is male.

**Table A6:** Analysis of association between individual risk factors and change in immediate recall between wave 1 and wave 4 for the 60s cohort

|  | Reg Coef | 95% CI | p-value |
| --- | --- | --- | --- |
| **Biomarker** |  |  |  |
| APOE e4 (-/-) | Ref |  |  |
| APOE e4 (-/+) | -0.12 | -0.34, 0.11 | 0.31 |
| APOE e4 (+/+) | **-1.06** | **-1.78, -0.34** | **<0.01** |
| Interaction (Male* APOE e4 (-/-)) | Ref |  |  |
| Interaction (Female* APOE e4 (-/+)) | -0.07 | -0.40, 0.25 | 0.66 |
| Interaction (Female*APOE e4 (+/+)) | **1.00** | **0.04, 1.96** | **0.04** |
| **Demographic** |  |  |  |
| Childhood financial hardship | 0.03 | -0.22, 0.27 | 0.84 |
| Interaction (Sex* Childhood financial hardship) | -0.29 | -0.67, 0.09 | 0.13 |
| Finance problem | **-0.40** | **-0.71, -0.08** | **0.02** |
| Interaction (Sex* Finance problem) | 0.24 | -0.20, 0.68 | 0.28 |
| Interaction (Sex* Finance problem* wave) | 0.03 | -0.81, 0.88 | 0.94 |
| Partnership status | -0.22 | -0.54, 0.10 | 0.18 |
| Interaction (Sex* Partnership status**)** | 0.16 | -0.23, 0.56 | 0.42 |
| Interaction (Sex* Partnership status*wave**)** | -0.08 | -0.57, 0.42 | 0.76 |
| **Lifestyle** |  |  |  |
| Currently smoking | -0.20 | -0.54, 0.14 | 0.24 |
| Interaction (Sex* Currently smoking**)** | -0.15 | -0.66, 0.35 | 0.55 |
| Interaction (Sex* Currently smoking*wave**)** | 0.40 | -0.48, 1.28 | 0.37 |
| Alcohol abstains | -0.10 | -0.47, 0.27 | 0.59 |
| Interaction (Sex* Alcohol abstains**)** | -0.39 | -0.85, 0.07 | 0.10 |
| Interaction (Sex* Alcohol abstains *wave**)** | 0.42 | -0.21, 1.05 | 0.19 |
| Moderate drinking | 0.14 | -0.10, 0.38 | 0.26 |
| Interaction (Sex* Moderate drinking**)** | 0.07 | -0.26, 0.39 | 0.69 |
| Interaction (Sex* Moderate drinking*wave**)** | -0.09 | -0.52, 0.35 | 0.70 |
| Moderate activity | **0.27** | **0.02, 0.52** | **0.03** |
| Interaction (Sex* Moderate activity**)** | 0.06 | -0.30, 0.43 | 0.73 |
| Interaction (Sex* Moderate activity*wave**)** | 0.11 | -0.41, 0.63 | 0.68 |
| Vigorous activity | **0.34** | **0.02, 0.66** | **0.03** |
| Interaction (Sex* Vigorous activity**)** | -0.16 | -0.68, 0.36 | 0.54 |
| Interaction (Sex* Vigorous activity*wave**)** | 0.12 | -0.67, 0.91 | 0.77 |
| Cognitive engagement | **0.31** | **-0.00, 0.62** | **0.05** |
| Interaction (Sex* Cognitive engagement**)** | 0.05 | -0.36, 0.46 | 0.80 |
| Interaction (Sex* Cognitive engagement *wave**)** | -0.00 | -0.57, 0.57 | 0.99 |
| Sleep problem | 0.14 | -0.09, 0.38 | 0.23 |
| Interaction (Sex*Sleep problem**)** | -0.11 | -0.44, 0.22 | 0.52 |
| Interaction (Sex*Sleep problem *wave**)** | 0.11 | -0.35, 0.57 | 0.64 |
| Overweight | 0.11 | -0.14, 0.36 | 0.39 |
| Interaction (Sex* Overweight**)** | -0.13 | -0.49, 0.24 | 0.50 |
| Interaction (Sex* Overweight *wave**)** | 0.13 | -0.38, 0.63 | 0.62 |
| Obese | 0.11 | -0.22, 0.44 | 0.51 |
| Interaction (Sex* Obese**)** | -0.21 | -0.66, 0.25 | 0.37 |
| Interaction (Sex* Obese *wave**)** | 0.11 | -0.50, 0.72 | 0.73 |
| Schuster-social support score | **0.02** | **0.00, 0.03** | **0.02** |
| Interaction (Sex* SS**)** | -0.01 | -0.03, 0.01 | 0.24 |
| Interaction (Sex* SS*wave**)** | 0.00 | -0.03, 0.03 | 0.97 |
| **Medical** |  |  |  |
| Arthritis | 0.01 | -0.23, 0.26 | 0.91 |
| Interaction (Sex* Arthritis**)** | -0.12 | -0.44, 0.21 | 0.47 |
| Interaction (Sex* Arthritis *wave**)** | 0.07 | -0.38, 0.53 | 0.75 |
| Cancer | 0.21 | -0.21, 0.63 | 0.33 |
| Interaction (Sex* Cancer**)** | 0.15 | -0.47, 0.77 | 0.64 |
| Interaction (Sex* Cancer *wave**)** | -0.05 | -0.75, 0.65 | 0.89 |
| Diabetes | -0.04 | -0.42, 0.33 | 0.81 |
| Interaction (Sex* Diabetes**)** | 0.02 | -0.57, 0.61 | 0.94 |
| Interaction (Sex* Diabetes *wave**)** | -0.36 | -1.06, 0.34 | 0.31 |
| Hypertension | 0.06 | -0.17, 0.30 | 0.58 |
| Interaction (Sex* Hypertension**)** | **-0.37** | **-0.70, -0.05** | **0.02** |
| Interaction (Sex Hypertension*wave**)** | 0.25 | -0.24, 0.73 | 0.31 |
| Stroke | 0.04 | -0.38, 0.47 | 0.85 |
| Interaction (Sex*Stroke**)** | -0.36 | -0.99, 0.27 | 0.27 |
| Interaction (Sex*Stroke*wave**)** | 0.37 | -0.46, 1.21 | 0.38 |
| Any head injury | 0.12 | -0.18, 0.41 | 0.44 |
| Interaction (Sex* Any head injury**)** | -0.19 | -0.68, 0.30 | 0.45 |
| Interaction (Sex* Any head injury *wave**)** | 0.32 | -0.29, 0.93 | 0.31 |
| Anxiety (Goldberg scales) | -0.01 | -0.09, 0.06 | 0.69 |
| Interaction (Sex* Anxiety**)** | -0.00 | -0.07, 0.06 | 0.95 |
| Interaction (Sex* Anxiety *wave**)** | -0.03 | -0.12, 0.07 | 0.61 |
| Depression (Goldberg scales) | -0.04 | -0.10, 0.02 | 0.16 |
| Interaction (Sex* Depression**)** | -0.05 | -0.14, 0.03 | 0.20 |
| Interaction (Sex* Depression *wave**)** | 0.05 | -0.06, 0.17 | 0.36 |
| **Pharmacological** |  |  |  |
| Anti-hypertensive | -0.17 | -0.40, 0.06 | 0.14 |
| Interaction (Sex* Anti-hypertensive**)** | 0.01 | -0.31, 0.34 | 0.94 |
| Interaction (Sex* Anti-hypertensive *wave**)** | -0.09 | -0.55, 0.36 | 0.68 |
| Cholesterol medication | 0.20 | -0.05, 0.44 | 0.11 |
| Interaction (Sex* Cholesterol med**)** | -0.28 | -0.65, 0.08 | 0.13 |
| Interaction (Sex* Cholesterol med*wave**)** | 0.18 | -0.28, 0.64 | 0.44 |
| Any Anti-inflammatory | -0.01 | -0.38, 0.35 | 0.95 |
| Interaction (Sex* Anti-inflammatory**)** | 0.14 | -0.34, 0.63 | 0.56 |

*Adjusted for age, race, non-English speaking background, year of education and wave. Reference group is male.

**Table A7:** Attrition by variables for the 40s and 60s cohorts

|  | **40s (n=2527)** | | | | **60s (n=2551)** | | | |
| --- | --- | --- | --- | --- | --- | --- | --- | --- |
|  | Wave 1 | Wave 2 | Wave 3 | Wave 4 | Wave 1 | Wave 2 | Wave 3 | Wave 4 |
| Cumulative # of death before interview | 0 | 3 | 16 | 40 | 0 | 122 | 223 | 351 |
| Childhood financial distress | 0.4% | --- | --- | --- | 0.1% | --- | --- | --- |
| ApoeE4 genotype | 9.1% | --- | --- | --- | 6.4% | --- | --- | --- |
| age | 0.0% | 6.9% | 13.3% | 27.4% | 0.0% | 7.9% | 14.5% | 24.5% |
| Year of education | 0.0% | 6.9% | 0.0% | 0.0% |  |  |  |  |
| Partnership status | 0.0% | 6.9% | 13.5% | 27.9% | 0.0% | 7.9% | 14.6% | 24.7% |
| Smoking status | 0.0% | 6.9% | 13.5% | 27.7% | 0.0% | 7.9% | 14.5% | 24.5% |
| Finance problem | 20.5% | 7.1% | 13.3% | 28.1% | 0.0% | 8.0% | 14.6% | 24.6% |
| Diabetes | 0.0% | 9.0% | 13.6% | 28.6% | 0.0% | 11.1% | 14.6% | 25.4% |
| Arthritis | 0.0% | 8.5% | 13.5% | 28.6% | 0.1% | 9.6% | 14.6% | 25.4% |
| Blood pressure medication | 0.0% | 7.0% | 13.5% | 28.6% | 0.1% | 8.0% | 14.6% | 25.5% |
| Cholesterol medication | NA | NA | NA | NA | 0.0% | 8.0% | 14.6% | 25.5% |
| Goldberg anxiety | 0.6% | 7.2% | 13.7% | 27.9% | 0.4% | 8.8% | 15.2% | 24.7% |
| Goldberg depression | 0.6% | 7.3% | 13.7% | 27.9% | 0.4% | 8.9% | 15.2% | 24.8% |
| Exercise categories | 7.1% | 8.4% | 13.9% | 44.8% | 11.9% | 11.4% | 15.3% | 37.3% |
| Hypertension | 1.3% | 9.5% | 16.2% | 37.1% | 1.3% | 9.3% | 16.3% | 27.7% |
| Cancer | 0.0% | 8.7% | 13.6% | 28.6% | 0.0% | 10.9% | 14.6% | 25.4% |
| Mental activity | 0.8% | 7.3% | 14.0% | 28.9% | 0.5% | 9.1% | 15.7% | 25.5% |
| BMI | 7.7% | 12.1% | 13.8% | 30.4% | 9.4% | 10.0% | 16.1% | 26.3% |
| Alcohol group | 0.0% | 6.9% | 13.5% | 27.9% | 0.1% | 8.0% | 14.7% | 24.7% |
| Sleep problem | 0.6% | 7.2% | 13.7% | 27.9% | 0.4% | 8.4% | 15.1% | 24.7% |
| Social support score | 0.4% | 28.2% | 33.6% | 28.6% | 1.0% | 30.3% | 48.4% | 28.5% |
| Immediate recall | 0.4% | 7.8% | 16.2% | 39.2% | 0.0% | 9.5% | 15.5% | 25.3% |
| Delayed recall | 0.4% | 7.8% | 16.2% | 39.3% | 0.0% | 9.5% | 15.6% | --- |
